# Insights into phylogenetic relationships and genome evolution of subfamily Commelinoideae (Commelinaceae Mirb.) inferred from complete chloroplast genomes

**DOI:** 10.1186/s12864-021-07541-1

**Published:** 2021-04-02

**Authors:** Joonhyung Jung, Changkyun Kim, Joo-Hwan Kim

**Affiliations:** 1 Department of Life Sciences, Gachon University, 1342 Seongnamdaero, Seongnam-si, Gyeonggi-do 13120 Republic of Korea; 2 Plant Research Division, Honam National Institute of Biological Resources, 99 Gohadoan-gil, Mokpo-si, Jeollanam-do 58762 Republic of Korea

**Keywords:** Commelinaceae, Chloroplast genome, Nucleotide diversity, Phylogenomics, Plastome

## Abstract

**Background:**

Commelinaceae (Commelinales) comprise 41 genera and are widely distributed in both the Old and New Worlds, except in Europe. The relationships among genera in this family have been suggested in several morphological and molecular studies. However, it is difficult to explain their relationships due to high morphological variations and low support values. Currently, many researchers have been using complete chloroplast genome data for inferring the evolution of land plants. In this study, we completed 15 new plastid genome sequences of subfamily Commelinoideae using the Mi-seq platform. We utilized genome data to reveal the structural variations and reconstruct the problematic positions of genera for the first time.

**Results:**

All examined species of Commelinoideae have three pseudogenes (*acc*D, *rpo*A, and *ycf*15), and the former two might be a synapomorphy within Commelinales. Only four species in tribe Commelineae presented IR expansion, which affected duplication of the *rpl*22 gene. We identified inversions that range from approximately 3 to 15 kb in four taxa (*Amischotolype*, *Belosynapsis*, *Murdannia*, and *Streptolirion*). The phylogenetic analysis using 77 chloroplast protein-coding genes with maximum parsimony, maximum likelihood, and Bayesian inference suggests that *Palisota* is most closely related to tribe Commelineae, supported by high support values. This result differs significantly from the current classification of Commelinaceae. Also, we resolved the unclear position of Streptoliriinae and the monophyly of Dichorisandrinae. Among the ten CDS (*ndh*H, *rpo*C2, *ndh*A, *rps*3, *ndh*G, *ndh*D, *ccs*A, *ndh*F, *mat*K, and *ycf*1), which have high nucleotide diversity values (Pi > 0.045) and over 500 bp length, four CDS (*ndh*H, *rpo*C2, *mat*K, and *ycf*1) show that they are congruent with the topology derived from 77 chloroplast protein-coding genes.

**Conclusions:**

In this study, we provide detailed information on the 15 complete plastid genomes of Commelinoideae taxa. We identified characteristic pseudogenes and nucleotide diversity, which can be used to infer the family evolutionary history. Also, further research is needed to revise the position of *Palisota* in the current classification of Commelinaceae.

**Supplementary Information:**

The online version contains supplementary material available at 10.1186/s12864-021-07541-1.

## Introduction

Commelinaceae Mirb., commonly known as the dayflower and spiderwort family, are the largest family of Commelinales Mirb. ex Bercht. & J. Presl, including four other families: Haemodoraceae, Hanguanaceae, Philydraceae, and Pontederiaceae, [1, 2]. Commelinaceae consist of 41 genera and approximately 730 species, widely distributed in both the Old and New Worlds, except in Europe [2–4]. Genus *Callisia* Loefl. and *Tradescantia* L. emend. M. Pell. are commonly used as ornamentals, while *Commelina* L. is used as vegetables and more commonly known as troublesome weeds. The species of Commelinaceae are usually succulent herbs with closed leaf-sheaths, raphide-canals, and three-celled glandular microhairs [3, 4]. Additionally, flowers of Commelinaceae are mainly insect-pollinated, have short blooming times, and lack any kind of nectaries [5, 6]. The flowering unit (inflorescence) of Commelinaceae is a many-branched thyrse, with each branch generally consisting of a many-flowered cincinnus. The cincinni can sometimes be 1-flowered or, more rarely, the whole inflorescence can be reduced to a single flower [4, 7].

Previous classifications of Commelinaceae emphasized floral and anatomical characters. In the first classification, Commelinaceae were divided into two tribes, Commelineae and Tradescantieae, based on the number of stamens and their fertility [8]. Then, Bruckner [9] used flower symmetry, and Pichon [10] used anatomical characters to exclude *Cartonema* R. Br. from Commelinaceae. In 1966, 15 genera of Commelinaceae were defined using various floral characters [11]. In the current classification, Commelinaceae were divided into two subfamilies, Cartonematoideae (Pichon) Faden ex G. C. Tucker and Commelinoideae Faden & D. R. Hunt, based on the presence of raphide-canals and glandular microhairs [4]. Cartonematoideae consists of two genera (*Cartonema* and *Triceratella* Brenan), whereas Commelinoideae includes 39 genera, divided into two tribes, Commelineae (Meisn.) Faden & D. R. Hunt and Tradescantieae (Meisn.) Faden & D. R. Hunt, based on palynological characters. The latter tribe was arranged into seven subtribes based on morphological and cytological characters [4, 12]. However, it is difficult to interpret relationships among genera due to their morphological variation. The morphology-based phylogeny was highly homoplasy and incongruent with the current classification [13]. In order to clarify the relationships within Commelinaceae, several phylogenetic studies have been conducted [14–20]. Based solely on the plastidial *rbc*L marker, *Cartonema* was recovered in a basal clade, and both Commelineae and Tradescantieae were monophyletic, except for the position of *Palisota* Rchb., which had low support values [15]. Furthermore, the plastidial *ndh*F suggested that subtribe Tradescantiinae was paraphyletic, whereas Thyrsantheminae and Dichorisandrinae were polyphyletic [16]. Combined data of nuclear 5S NTS and plastid *trn*L-F regions resulted in a well-supported relationship between Commelineae and Tradescantieae. However, the position of *Palisota* and *Spatholirion* Ridl. were ambiguous [17].

Chloroplast genome or plastid genome (cpDNA) is highly conserved and has a typical quadripartite structure containing a large single copy (LSC) and a small single copy (SSC) separated by two inverted repeats (IRs). The size of cpDNA ranges from 19,400 bp (*Cytinus hypocistis*) to 242,575 bp (*Pelargonium transvaalense*) and generally contains 120–130 genes, which perform important roles in photosynthesis, translation, and transcription [21, 22]. The rapid development of next-generation sequencing (NGS) has enabled many studies with high-quality complete plastid genomes with raw reads at low costs. Due to its conserved characteristics, chloroplast protein-coding genes were used to reconstruct the phylogenetic relationships in other monocot groups [23–25]. Furthermore, these data are useful to infer biogeography, molecular evolution, and age estimation [26–28]. The aims of this study are to 1) explore the genome evolution in Commelinaceae subfamily Commelinoideae through analyses of sequence variation, and gene content and order; 2) find latent phylogenetically informative genes through high nucleotide diversity; 3) reconstruct the phylogenetic relationships among members of Commelinoideae with other monocot groups using 77 chloroplast protein-coding genes data, especially the relationships among the six subtribes of Tradescantieae.

## Results

### Chloroplast genome assembly and annotation

**Fig. 1:**
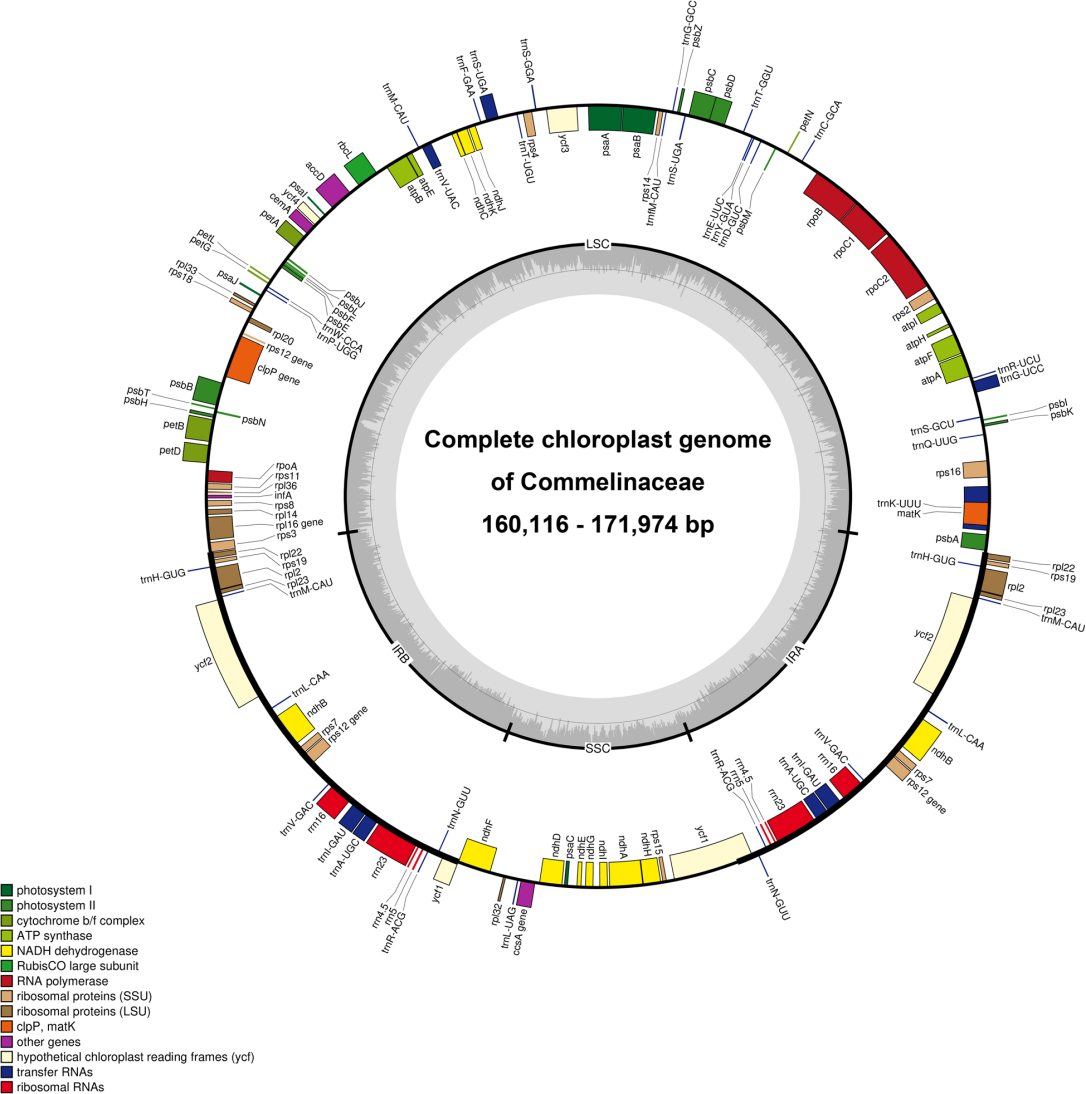
Representative chloroplast genome of Commelinaceae. The colored boxes represent conserved chloroplast genes. Genes shown inside the circle are transcribed clockwise, whereas genes outside the circle are transcribed counter-clockwise. The small grey bar graphs inner circle shows the GC contents

We completed 15 new plastid genomes in this study listed in Table 1 through 9 to 21 million raw reads for each species (Fig. S1, Table S1). A total of 16 plastid genomes, including *Belosynapsis ciliata*, exhibit the typical quadripartite structure containing LSC and SSC regions separated by two inverted repeats (Fig. 1). Plastid genome sequences of *Murdannia edulis* and *B. ciliata* are over 170 kb in length whereas that of *Commelina communis* is 160,116 bp in length (Table 1). In addition, *M. edulis* has the lowest GC content (34.4%), whereas *Palisota barteri* has the highest GC content (36.2%) (Table 1). The highest length difference (about 8801 bp) was observed in the LSC region, between *B. ciliata* and *C. communis*. GC content in the SSC region was about 3.4% between *Dichorisandra thyrsiflora* and *M. edulis* (Table 1). Plastid genomes of Commelinoideae have 131 genes, of which 111 are unique, and 20 are duplicated in the IR regions (Table 2), except for the *rpl*22 gene, which was not duplicated in tribe Tradescantieae. There are 77 protein-coding genes (CDS), 30 transfer RNA (tRNA) genes and four ribosomal RNA (rRNA) genes in examined Commelinoideae taxa (Table 2). In these genes, three CDS (*rps*12, *clp*P, and *ycf*3) have two introns, while nine CDS (*atp*F, *ndh*A, *ndh*B, *pet*B, *pet*D, *rpl*2, *rpl*16, *rpo*C1, and *rps*16) and six tRNA (*trn*K-UUU, *trn*G-UCC, *trn*L-UAA, *trn*V-UAC, *trn*I-GAU, and *trn*A-UGC) have one intron (Table 2). The *rps*12 gene was trans-spliced, which has the 5′ exon in the LSC region and the 3′ exon and intron in the IR regions. Three pseudogenes (*acc*D, *rpo*A, and *ycf*15) were identified from all Commelinoideae species, one (*ycf*15) of which was duplicated in the IR regions (Table 2). These three genes contained several internal stop codons due to insertions and deletions, thus are identified as pseudogenes. Also, we identified *ndh*B as a pseudogene in two species (*Pollia japonica* and *Rhopalephora scaberrima*) due to point mutation.

**Table 1:** Comparison of the features of plastomes from 16 genera of Commelinaceae

Taxa	Tribe	Subtribe	Length and G + C content				GenBank accession number	Voucher
			LSC bp (G + C%)	SSC bp (G + C%)	IR bp (G + C%)	Total bp (G + C%)		
*Gibasis geniculata*	Tradescantieae	Tradescantiinae	89,154(33.3)	18,278(30.5)	26,953(42.5)	161,338(36.1)	MW617987	JH200402001
*Tradescantia virginiana*	Tradescantieae	Tradescantiinae	91,991(32.7)	18,462(30.2)	27,236(42.3)	164,925(35.6)	MW617994	JH170813001
*Callisia repens*	Tradescantieae	Tradescantiinae	89,446(33.2)	18,252(30.3)	27,078(42.5)	161,854(36.0)	MW617982	JH190318001
*Weldenia candida*	Tradescantieae	Tradescantiinae	95,029(32.6)	19,024(30.3)	27,233(42.6)	168,519(35.5)	MW617995	JH190730001
*Amischotolype hispida*	Tradescantieae	Coleotrypinae	94,525(32.9)	19,255(30.4)	27,385(42.4)	168,550(35.7)	MW617981	JH191109002
*Belosynapsis ciliata*	Tradescantieae	Cyanotinae	96,164(31.3)	20,224(28.0)	27,241(42.6)	170,870(34.5)	MK133255.1	–
*Cochliostema odoratissimum*	Tradescantieae	Dichorisandrinae	92,560(33.2)	18,856(30.4)	27,276(42.5)	165,968(35.9)	MW617983	JH190310001
*Geogenanthus poeppigii*	Tradescantieae	Dichorisandrinae	94,583(32.8)	18,612(30.7)	27,098(42.5)	167,391(35.7)	MW617986	JH190803001
*Dichorisandra thyrsiflora*	Tradescantieae	Dichorisandrinae	94,347(32.9)	18,348(31.1)	27,194(42.6)	167,083(35.8)	MW617985	JH190616001
*Siderasis fuscata*	Tradescantieae	Dichorisandrinae	94,389(32.9)	18,606(31.0)	27,196(42.6)	167,387(35.8)	MW617992	XX-0-GENT-19822394
*Streptolirion volubile*	Tradescantieae	Streptoliriinae	91,528(33.1)	19,595(29.3)	27,447(42.0)	166,017(35.6)	MW617993	JH180919003
*Palisota barteri*	Tradescantieae	Palisotinae	93,315(33.5)	18,905(30.8)	27,074(42.7)	166,368(36.2)	MW617989	JH190222001
*Pollia japonica*	Commelineae	–	90,295(33.2)	19,151(29.7)	27,604(42.2)	164,654(35.8)	MW617990	JH180805001
*Rhopalephora scaberrima*	Commelineae	–	87,602(33.2)	18,354(29.5)	27,487(42.1)	160,930(35.8)	MW617991	JH191109014
*Commelina communis*	Commelineae	–	87,363(33.0)	18,561(29.1)	27,096(42.3)	160,116(35.7)	MW617984	JH180709001
*Murdannia edulis*	Commelineae	–	96,248(31.4)	20,798(27.7)	27,464(42.1)	171,974(34.4)	MW617988	JH191110010

**Table 2:** Gene composition within chloroplast genomes of Commelinaceae species

Groups of genes		Names of genes	No.
RNA genes	Ribosomal RNAs	*rrn*4.5 ^X2^, *rrn*5 ^X2^, *rrn*16 ^X2^, *rrn*23 ^X2^	8
	Transfer RNAs	*trnK*-UUU ^a^, *trnQ-*UUG, *trnS*-GCU, *trnG*-UCC ^a^, *trnR*-UCU, *trnC*-GCA, *trnD*-GUC, *trnY*-GUA, *trnE*-UUC, *trnT*-GGU, *trnS*-UGA, *trnG*-GCC, *trnfM*-CAU, *trnS*-GGA*, trnT*-UGU, *trnL*-UAA ^a^, *trnF*-GAA, *trnV*-UAC^a^, *trnM*-CAU, *trnW*-CCA, *trnP*-UGG, *trnH*-GUG ^X2^, *trnI*-CAU ^X2^, *trnL*-CAA ^X2^, *trnV*-GAC ^X2^, *trnI*-GAU ^a X2^, *trnA*-UGC ^a X2^, *trnR*-ACG ^X2^, *trnN*-GUU ^X2^, *trnL*-UAG	38
Protein genes	Photosystem I	*psa*A, *psa*B, *psa*C, *psa*I, *psa*J	5
	Photosystem II	*psb*A, *psb*B, *psb*C, *psb*D, *psb*E, *psb*F, *psb*H, *psb*I, *psb*J, *psb*K, *psb*L, *psb*M, *psb*N, *psb*T, *psb*Z	15
	Cytochrome	*pet*A, *pet*B ^a^, *pet*D ^a^, *pet*G, *pet*L, *pet*N	6
	ATP synthases	*atp*A, *atp*B, *atp*E, *atp*F ^a^, *atp*H, *atp*I	6
	Large unit of Rubisco	*rbc*L	1
	NADH dehydrogenase	*ndh*A ^a^, *ndh*B ^a X2^, *ndh*C, *ndh*D, *ndh*E, *ndh*F, *ndh*G, *ndh*H, *ndh*I, *ndh*J, *ndh*K	12
	ATP-dependent protease subunit P	*clp*P ^b^	1
	Envelope membrane protein	*cem*A	1
Ribosomal proteins	Large units of ribosome	*rpl*2 ^a X2^, *rpl*14, *rpl*16 ^a^, *rpl*20, *rpl*22 ^X2^, *rpl*23 ^X2^, *rpl*32, *rpl*33, *rpl*36	12
	Small units of ribosome	*rps*2, *rps*3, *rps*4, *rps*7 ^X2^, *rps*8, *rps*11, *rps*12 ^X2^, *rps*14, *rps*15, *rps*16 ^a^, *rps*18, *rps*19 ^X2^	15
Transcription/translation	RNA polymerase	*rpo*A^⍦^, *rpo*B*, rpo*C1 ^a^, *rpo*C2	3
	Initiation factor	*inf*A	1
	Miscellaneous protein	*acc*D^⍦^, *ccs*A, *mat*K	2
	Hypothetical proteins and conserved reading frames	*ycf*1, *ycf*2 ^X2^, *ycf*3 ^b^, *ycf*4, *ycf*15^⍦^	5
Total			131

^a^gene with one intron; ^b^gene with two introns; X2: duplicated gene; ⍦: pseudogene

### Comparative chloroplast genome structure and nucleotide diversity

**Fig. 2:**
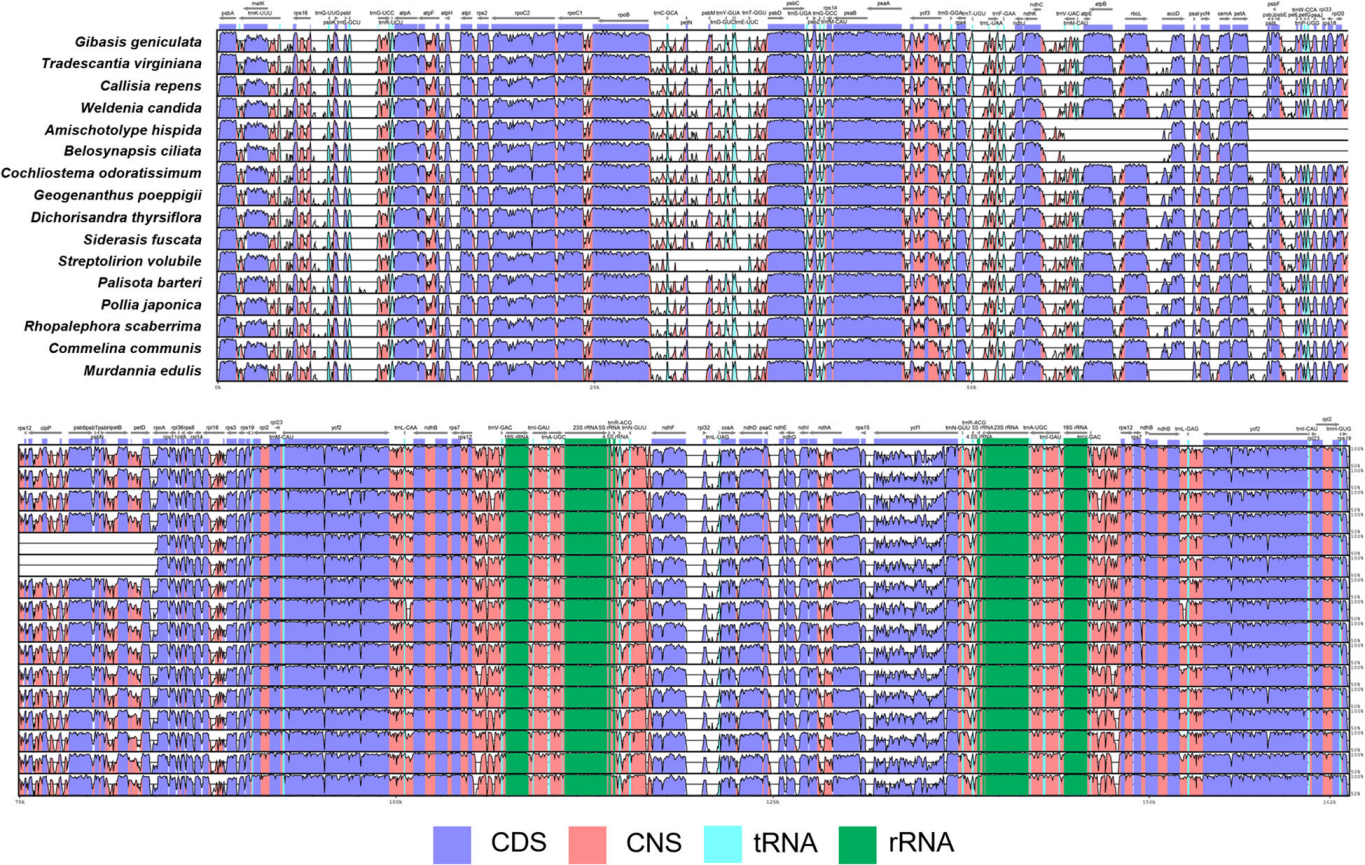
Plots of percent sequence identity of the chloroplast genomes of 16 Commelinaceae species with *Hanguana malayana* as a reference. The percentage of sequence identities was estimated, and the plots were visualized in mVISTA

**Fig. 3:**
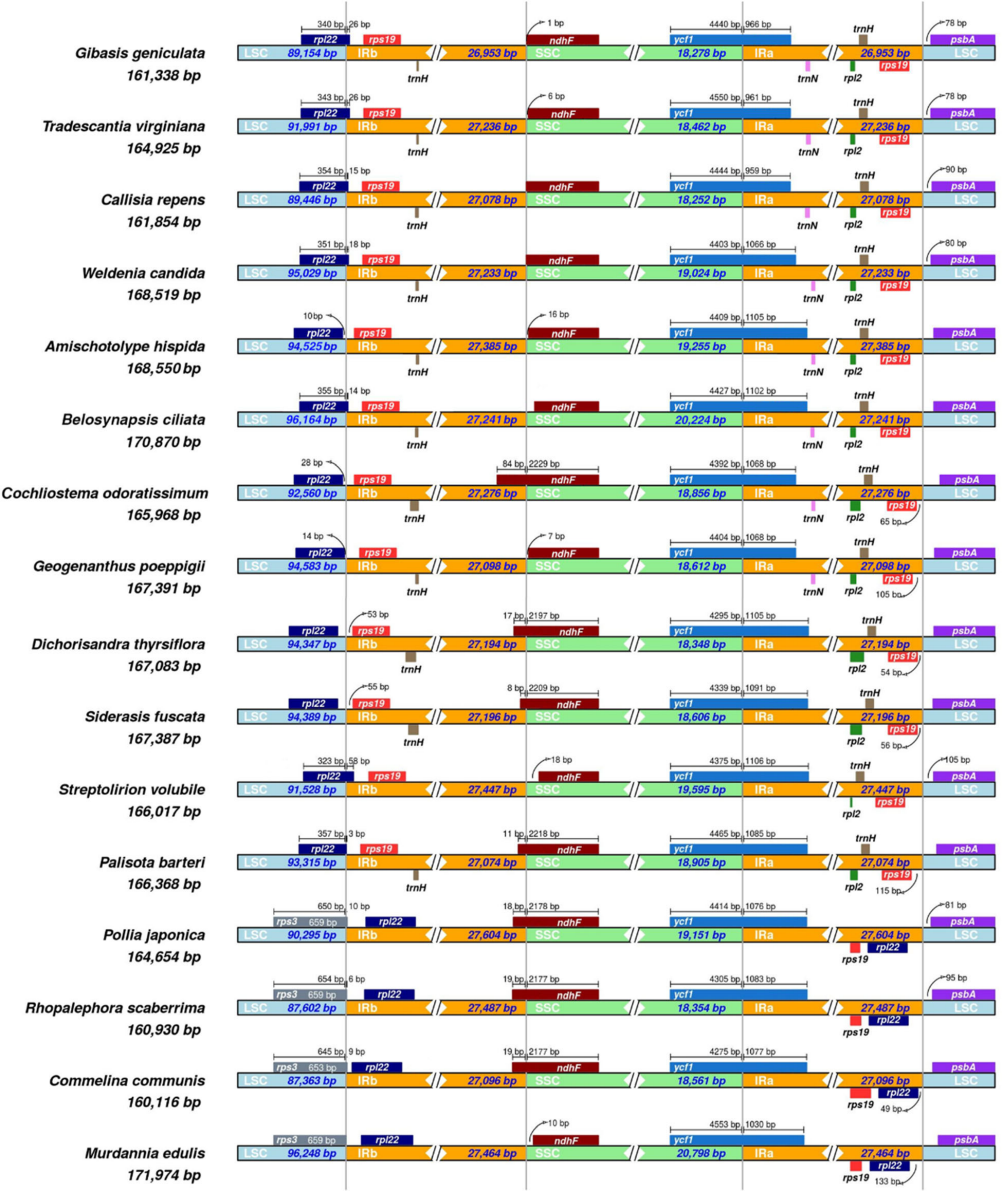
Comparisons of LSC, SSC, and IR regions boundaries between 16 Commelinaceae species

The aligned data of whole plastid genomes showed high similarities in coding genes, and high variations in non-coding genes (Fig. 2). We found several genome structure variations among Commelinoideae species. *M. edulis* and *Streptolirion volubile* had one inversion from *rbc*L to *psa*I intergenetic spacer (approximately 3 kb) and *pet*N to *trn*E-UUC (approximately 2.8 kb), respectively. *Amischotolype hispida* and *B. ciliata* had two large inversions from *trn*V-UAC to *rbc*L and *psb*J to *pet*D about approximately 5 kb and 16 kb, respectively. The IR-SSC boundary was similar among species of Commelinoideae (Fig. 3). All plastid genomes have an incompletely duplicated *ycf*1 gene in the IR_B_-SSC junctions. We also found an expansion of IR regions in tribe Commelineae, which resulted in the duplication of the *rpl*22 genes (Fig. 3).

**Fig. 4:**
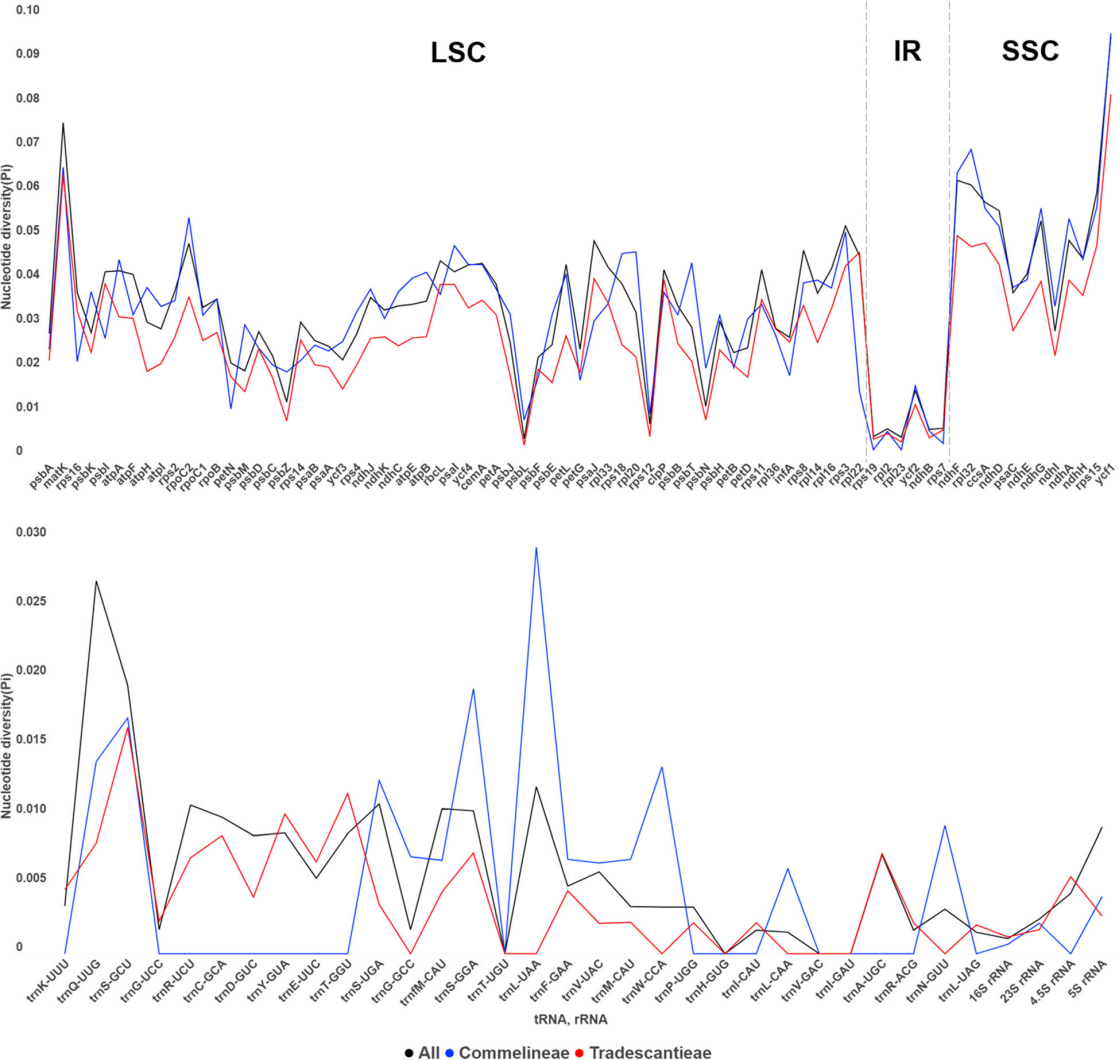
Nucleotide diversity (Pi) values in protein-coding genes, tRNA, and rRNA in 16 Commelinaceae species. The dashed lines are the borders of the LSC, IR and SSC regions

**Fig. 5:**
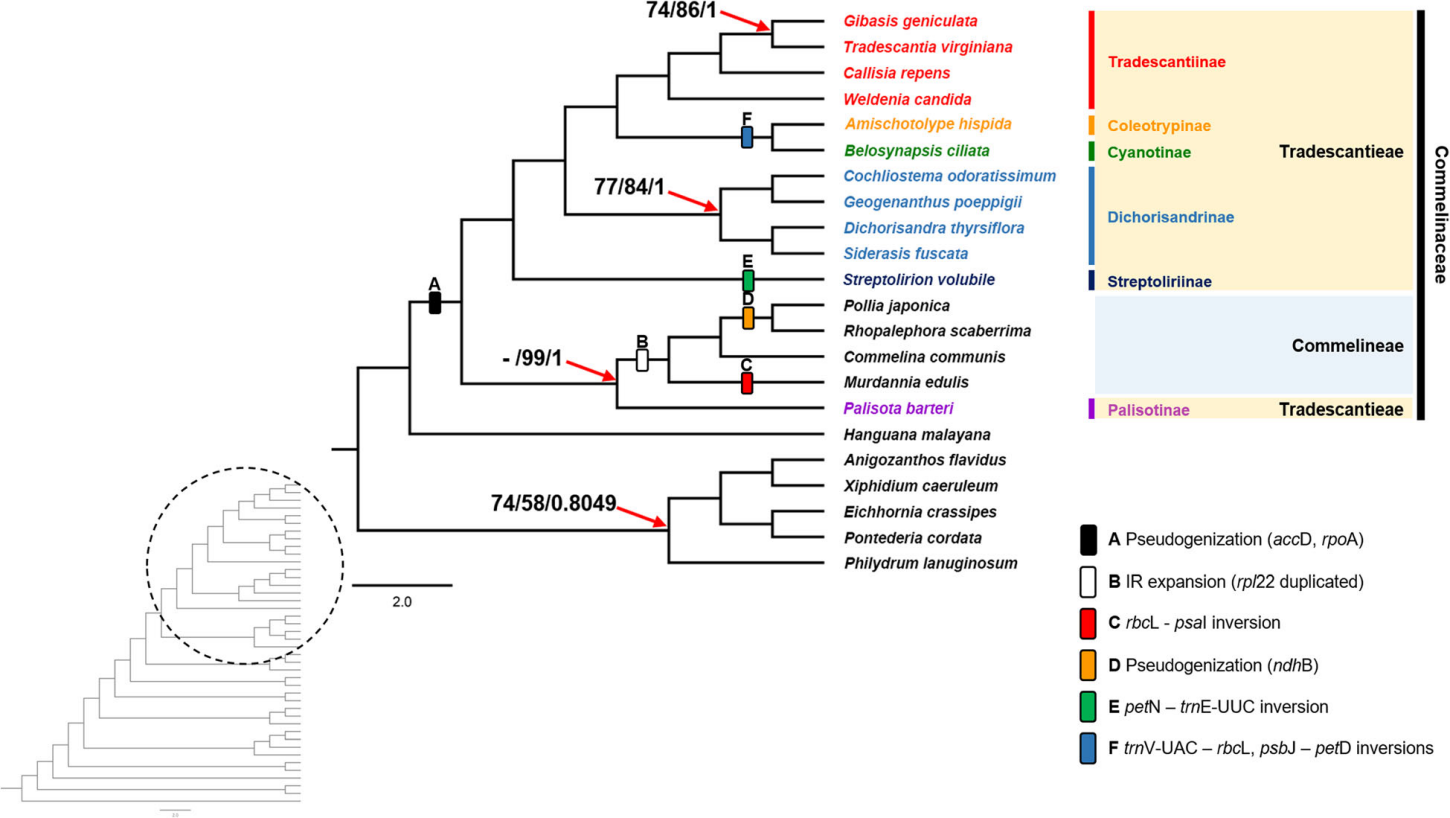
The Maximum Likelihood tree of 42 monocots inferred from 77 chloroplast protein-coding genes. Numbers indicate support (maximum parsimony bootstrap (PBP)/maximum likelihood bootstrap (MBP)/posterior probability (PP)). Only support under PBP = 90/MBP = 100/PP = 1.00 is shown. The dashes “-” indicate incongruence between MP and ML/BI trees

We analyzed nucleotide divergences of CDS, tRNA, and rRNA to explain variant characteristics among the 16 Commelinoideae plastid genomes (Fig. 4, Table S3). Nucleotide diversity (Pi) for each CDS ranges from 0.00427 (*psb*L) to 0.09543 (*ycf*1) with an average of 0.03473. Nine CDS (*rps*3, *ndh*G, *ndh*D, *ccs*A, *rps*15, *rpl*32, *ndh*F, *mat*K, and *ycf*1) have remarkably high values (Pi > 0.05) and seven CDS (*psb*L, *rpl*23, *rps*19, *ndh*B, *rpl*2, *rps*7, *rps*12) have low values (Pi < 0.01; Fig. 4). Compared with tribe Tradescantieae, Commelineae have higher values in 59 out of 77 CDS (Fig. 4). The *rpl*22 gene has the highest difference of values between Commelineae (Pi = 0.01499) and Tradescantieae (Pi = 0.04655). In the tRNA and rRNA regions, Pi values range from 0 (*trn*T-UGU, *trn*H-GUG, *trn*V-GAC, and *trn*I-GAU) to 0.02697 (*trn*Q-UUG), with an average of 0.006. Commelineae has the highest value in the *trn*L-UAA (Pi = 0.02941), while Tradescantieae has no value in this gene. We tried to find latent phylogenetically informative genes for the Commelinoideae by checking individual CDS with high values (Pi > 0.045) and over 500 bp length. Ten CDS (*ndh*H, *rpo*C2, *ndh*A, *rps*3, *ndh*G, *ndh*D, *ccs*A, *ndh*F, *mat*K, and *ycf*1) were checked with a ML analysis and compared positions among 16 genera of Commelinoideae (Fig. 5). Four CDS (*ndh*H, *rpo*C2, *mat*K, and *ycf*1) have similar topology in Commelinoideae even though the other monocot groups were unclear.

### Phylogenetic analysis

The aligned 77 chloroplast protein-coding genes had 65,481 bp, of which 16,380 were parsimony informative. The MP analysis produced single most-parsimonious tree (tree length = 72,586, CI = 0.488, RI = 0.626). The tree topologies of the MP, ML, and BI analyzes were found to be congruent with 100% bootstrap (PBP, MBP) values and 1.00 Bayesian posterior probabilities (PP) in almost all nodes, except for *Palisota*, which was unresolved in MP analysis (not shown) (Fig. 5). The result suggested that *Palisota* was sister to the group consisting of the rest of Commelinoideae (Fig. 5). In Tradescantieae, Streptoliriinae was positioned at the basal node. Then, Dichorisandrinae divided into two clades ((*Dichorisandra*, *Siderasis*), (*Cochliostema*, *Geogenanthus*)) with relatively low support values in both MP and ML analysis (PBP = 77, MBP = 84, PP = 1) (Fig. 5). Among the remaining three subtribes, where two clades ((Coleotrypinae and Cyanotinae), (Tradescantiinae)) were formed with high support values (PBP = 100, MBP = 100, PP = 1), respectively (Fig. 5).

## Discussion

### Chloroplast genome structure

In this study, we completed 15 new plastid genomes of Commelinoideae taxa (Table 1). Plastid genomes have typical quadripartite structures, including LSC, SSC and two IR regions. Plastid genomes of Commelinoideae have variable total length and GC content. The LSC and SSC regions are relatively longer and higher AT-content than the IR region (Table 1). The functions of AT-rich sequences in the plastid genome were known as enhancing gene transfer success by making stable transcripts [29]. However, AT-rich sequences caused structural variations like inversions by their weak hydrogen bonding. In this study, we identified small to large inversions in four species (Fig. 2). There is one inversion in *M. edulis* and *S. volubile*, and two inversions in *A. hispida* and *B. ciliata* (Fig. 2). Inversions are known as common genomic rearrangement events and provide informative infrageneric relationships. In previous studies, inversions were caused by microhomology-driven recombination via short repeats and suggested the monophyly of tribe Desmodieae (Fabaceae) [30]. Our results also suggest that both *Amischotolype* and *Belosynapsis* have two large inversions in the same loci and formed a clade sister to subtribe Dichorisandrinae (Fig. 5).

We identified an IR expansion in members of Commelineae (*Commelina*, *Murdannia*, *Pollia*, and *Rhopalephora*). Four species have one more *rpl*22 gene, which is duplicated in the terminal IR regions (Fig. 3). Although IR expansion affected gene composition, the IR region’s total length is similar among 16 Commelinoideae species. IR expansion and contraction are important events in several families. In Ranunculaceae, IR expansion was detected as a synapomorphy of tribe Anemoneae [31]. Likewise, IR expansion lent further support to the relationship between two subfamilies Ehrhartoideae and Pooideae (Poaceae) [32]. This event also may be phylogenetically informative in Commelinoideae since only members of tribe Commelineae sharing this genome variation (Fig. 5).

Within Commelinoideae plastid genomes, three protein-coding genes (*acc*D, *rpo*A, and *ycf*15) were classified as pseudogenes (Fig. S2). The *ycf*15 gene has several abnormal stop codons caused by insertions and deletions (indel) of bases similar to other monocots. We also identified that all examined species have indels at the frontal part of the *acc*D gene (until 400 bp) and the terminal part of the *rpo*A gene (after 700 bp; Fig. S2). The *acc*D gene, encoding the beta-carboxyl transferase subunit of acetyl-CoA carboxylase, is found in most flowering plants and synthesizes fatty acids within the chloroplast. It was suggested as an essential gene associated with maintaining chloroplast structure [33]. However, it was reported as a gene loss or pseudogenization in Acoraceae and Poaceae [34, 35]. Recent studies suggested that the *acc*D gene was found to be nuclear originated in several eudicots [36, 37]. The *rpo*A gene, which encodes the alpha subunit of RNA polymerase, is also found in most flowering plants but was recorded to having been lost in the chloroplast genome of mosses [38]. In one species, *Physcomitrella patens* (Funariaceae), the *rpo*A gene was transferred to the nucleus [39]. We need further studies to confirm whether these two genes have been transferred to the nucleus or not in Commelinaceae. We identified that the pseudogened *acc*D and *rpo*A only appeared in Commelinoideae among Commelinales. It might be a specific character of gene composition in Commelinales. We also found a point mutated base in the third codon of the *ndh*B gene in *P. japonica* and *R. scaberrima*, which formed a clade in this study (Fig. 5).

We measured the nucleotide diversity of CDS, tRNA, and rRNA to identify the genetic divergence between 16 Commelinoideae plastid genomes. We found that the CDS in the IR regions have lower nucleotide diversity than that of the LSC and SSC regions (Fig. 4). This result has also been identified in the other monocots [40–42]. It is possibly attributed to a copy correction of the IR regions via gene conversion [43]. Especially, we can see this result in the *rpl*22 gene. Only Commelineae species present a duplicated *rpl*22 gene due to the above-mentioned IR expansion, while the remaining 12 taxa have one gene in the LSC or LSC-IR junction (Fig. 3). Difference of nucleotide diversity in this gene between Commelineae (Pi = 0.015) and Tradescantieae (Pi = 0.0466) is 0.0316. It might be phylogenetically useful information for Tradescantieae only.

### Implications of plastomes data for phylogenetic reconstructions

The first phylogenetic analysis of Commelinaceae based on *rbc*L marker revealed a relationship of 32 species representing 30 genera of Commelinaceae [15]. Cartonematoideae was in a basal clade, sister to Commelinoideae and all remaining species [15]. Aside from *Palisota*, Commelinoideae was divided into two tribes, Commelineae and Tradescantieae, with low bootstrap support values due to insufficient information [15]. Although several phylogenetic studies were conducted, the relationships between the genera of Commelinaceae have remained unresolved. The position of *Palisota* had been problematic, being recovered as: 1) sister to all genera of Commelinoideae with high bootstrap values [15]; 2) low bootstrap support value with other members of Tradescantieae [16]; or 3) sister to tribe Commelineae [19]. Subtribe Streptoliriinae was recovered as sister to tribe Commelineae in the *trn*L-*trn*F analysis [17]. Finally, subtribe Dichorisandrinae seemed polyphyletic in the previous studies [15, 16, 19, 44]. These results are most likely due to limited taxon sampling and/or used few informative genetic markers. The aligned 77 chloroplast coding genes in this study suggest a more well-supported relationship between the genera (Fig. 5). We identified that Commelinoideae divided into two clades, tribe Commelineae and Tradescantieae, with high support values (Fig. 5). However, *Palisota*, which belongs to Tradescantieae in the current classification [3], is recovered by us as sister to tribe Commelineae (Fig. 5). The ML and BI results present high support values, even though this relationship is unresolved in MP (data not shown). Compared with the current classification, it seems like that subsidiary cells in the stomata and exine morphology are homoplastic to divide two tribes in Commelinoideae [3]. In the Commelinaceae, *Palisota* is unusual genus for its unique morphological characters like a fleshy berry as a fruit, stamen and staminode arrangement, complex reproductive system, and a basic number of chromosome (*x* = 20) [13, 45]. Zygomorphic androecium character places *Palisota* within the Commelineae clade in the morphological cladistic analysis [13]. However, this character is also homoplastic within Commelinoideae. Further research is needed to suggest appropriate characters for *Palisota*. The four species of Commelineae sampled by us are recovered with a relationship similar to previous studies [15]: (*Murdannia*, (*Commelina*, (*Pollia*, *Rhopalephora*))). Within Tradescantieae, Streptoliriinae diverged first, followed by Dichorisandrinae divided into two clades with relatively low support values (PBP = 77/MBP = 84/PP = 1) (Fig. 5). The clade composed by Coleotrypinae and Cyanotinae is recovered following the diversion of subtribe Dichorisandrinae, which is sister to Tradescantiinae *sensu* Pellegrini, [46]. Interestingly, the Asian and African subtribe Coleotrypinae and Cyanotinae were nested well within the New World subtribes (Fig. 5). This result is similar to previous studies and support the hypothesis that one shift from the Old World to the New World followed by dispersal back to the Old World [15, 16].

## Conclusions

Our study revealed genome structural characteristics, nucleotide diversity, improved relationships between genera using 15 newly complete chloroplast genomes of Commelinoideae. Compared with other Commelinales, we found two characteristic pseudogenes in all members of Commelinoideae, which might be a synapomorphy within the order. We also reconstruct the phylogenetic relationships using 77 chloroplast protein-coding genes. Although not being able to address the Commelinaceae as a whole, due to not sampling of subfamily Cartonematoideae, we have been able to recover well-supported relationships for the taxa of Commelinoideae, especially between the subtribes of Tradescantieae. One interesting result was that *Palisota* (subtribe Palisotinae) is more closely related to tribe Commelineae than the remaining members of tribe Tradescantieae. In the current classification, *Palisota* is a member of Tradescantieae according to the number of subsidiary cells in stomata and pollen exine lacking spines [3]. However, it seems like that these characters are homoplastic, so we need a further study to suggest appropriate characters for two tribes in Commelinoideae. We resolved the ambiguous position of Streptoliriinae which was placed with Commelineae group [17]. Also, Dichorisandrinae was monophyletic in this study which was polyphyletic in the previous studies [15, 16, 19]. Four genes (*ndh*H, *rpo*C2, *mat*K, and *ycf*1) are congruent with the tree estimated from the 77 protein-coding genes. These genes will be helpful to reconstruct relationships of the whole Commelinaceae in the future. Future studies might use the information of chloroplast genomes, relationship between genera to define new classification of Commelinaceae that we provided in this study. These data will make sure the historical biogeography and genome evolution of Commelinaceae.

## Materials and methods

### Taxon sampling and DNA extraction

Fresh leaf samples were collected in the field and dried directly with silica gel in room temperature until DNA extraction (Table 1). The samples covered four out of 14 genera in tribe Commelineae and 11 out of 25 genera, including six subtribes of tribe Tradescantieae. We prepared the voucher specimens for all used samples and deposited them in the Gachon University Herbarium (GCU) with their accession numbers. We used a modified CTAB method to extract total DNA [47] and checked quality using a spectrophotometer (Biospec-nano; Shimadzu) and assessed by agarose gel electrophoresis.

### Genome sequencing, assembly, and annotation

Next-generation sequencing (NGS) was conducted using the Illumina MiSeq sequencing system (Illumina, Seoul, Korea). We imported NGS raw data and trimmed the ends limited to a 5% error probability to remove poor quality reads using Geneious prime 2020.1.2 [48]. Then, we performed ‘map to reference’ using the *Hanguana malayana* chloroplast genome (GenBank accession = NC_029962.1) as a reference to isolate cpDNA reads. De novo assembly was implemented to reassemble reads using Geneious prime 2020.1.2 [48]. We used newly generated sequences as a reference to reassemble raw reads. We repeated this step until quadripartite structures were completed. Gaps were filled by Sanger sequencing using specific primers. Gene content and order were annotated using *H. malayana* as a reference using 80% similarity to identify genes in Geneious. All tRNAs were checked by tRNAScan-SE [49] with default search mode. Illustrations of plastomes were produced using OGDraw [50].

### Comparative genome analysis

We compared genome structure, size, gene content across all 16 species including *B. ciliata* (GenBank accession = MK133255.1), subtribe Cyanotinae. The GC content was calculated and compared using Geneious. The whole chloroplast genome sequences of Commelinoideae species were aligned using MUSCLE embedded in Geneious and visualized using LAGAN mode in mVISTA [51, 52]. For the mVISTA plot, we used the annotated cpDNA of *H. malayana* as a reference. We also examined the nucleotide diversity (Pi) of chloroplast protein-coding genes, transfer RNA genes and ribosomal RNA genes among the 16 Commelinoideae species through a sliding window analysis using DnaSP v. 6.0 [53]. For the sequence divergence analysis, we applied the window size of 100 bp with a 25 bp step size. The IR and SC boundaries of the 16 Commelinoideae species were compared and illustrated using IRscope [54].

### Phylogenetic analysis

A total of 42 chloroplast genome sequences (including 15 new chloroplast genomes of Commelinoideae) were used (Table S2). We extracted 77 protein-coding genes and aligned them using the MUSCLE embedded in Geneious prime 2020.1.2 [48]. For the data set, *Acorus calamus* (Acoraceae) was designated as an outgroup. We performed maximum parsimony (MP), maximum likelihood (ML), and Bayesian inference (BI) to infer relationships of Commelinoideae and related taxa. The MP analyses were carried out in PAUP* v4.0a [55] with all characters equally weighted and unordered. Gaps were treated as missing data. Searches of 1000 random taxon addition replicates used tree-bisection-reconnection (TBR) branch swapping, and MulTrees permitted ten trees to be held at each step. Bootstrap analyses (PBP, parsimony bootstrap percentages, 1000 pseudoreplicates) were conducted to examine internal support with the same parameters. We used jModelTest version 2.1.7 [56, 57] to find the best model with Akaike’s information criterion (AIC) before running the ML and BI analyses. The GTR + I + G was the best model for the concatenated data sets. We used the IQ-TREE web server (http://iqtree.cibiv.univie.ac.at/) to make the ML searches [58]. Support value (MBP, mean bootstrap percentage) was calculated with 1000 replicates of ultrafast bootstrap [59]. MrBayes v3.2.7 [60] was used for BI analyses. Two simultaneous runs were performed starting from random trees for at least 1,000,000 generations. One tree was sampled every 1000 generations. In total, 25% of trees were discarded as burn-in samples. The remaining trees were used to construct a 50% majority-rule consensus tree, with the proportion bifurcations found in this consensus tree given as posterior probability (PP) to estimate the robustness of half of the BI tree. The effective sample size values (ESS) were then checked for model parameters (at least 200). The phylogenetic trees were edited using FigTree v1.4.4 program [61].

## Supplementary Information

**Additional file 1: Table S1.** List of sampling taxa from 15 species of Commelinaceae and assembly information. **Table S2.** List of species used for phylogenomic analyses. **Table S3.** Nucleotide diversity (Pi) of 16 Commelinoideae species. **Figure S1.** Complete chloroplast genome of 15 Commelinaceae taxa in this study. **Figure S2.** Amino acid alignment of plastid *acc*D and *rpo*A genes within 22 Commelinales taxa.

## Data Availability

The 15 chloroplast genomes sequences we obtained from this study were archived in NCBI. The accession numbers are presented in Table 1.
