# Supplementary figures and images for: Insights into phylogenetic relationships and genome evolution of subfamily Commelinoideae (Commelinaceae Mirb.) inferred from complete chloroplast genomes
Source: BMC Genomics. 2021 Apr 2;22:231. doi: 10.1186/s12864-021-07541-1 (PMC8017861; doi:10.1186/s12864-021-07541-1)

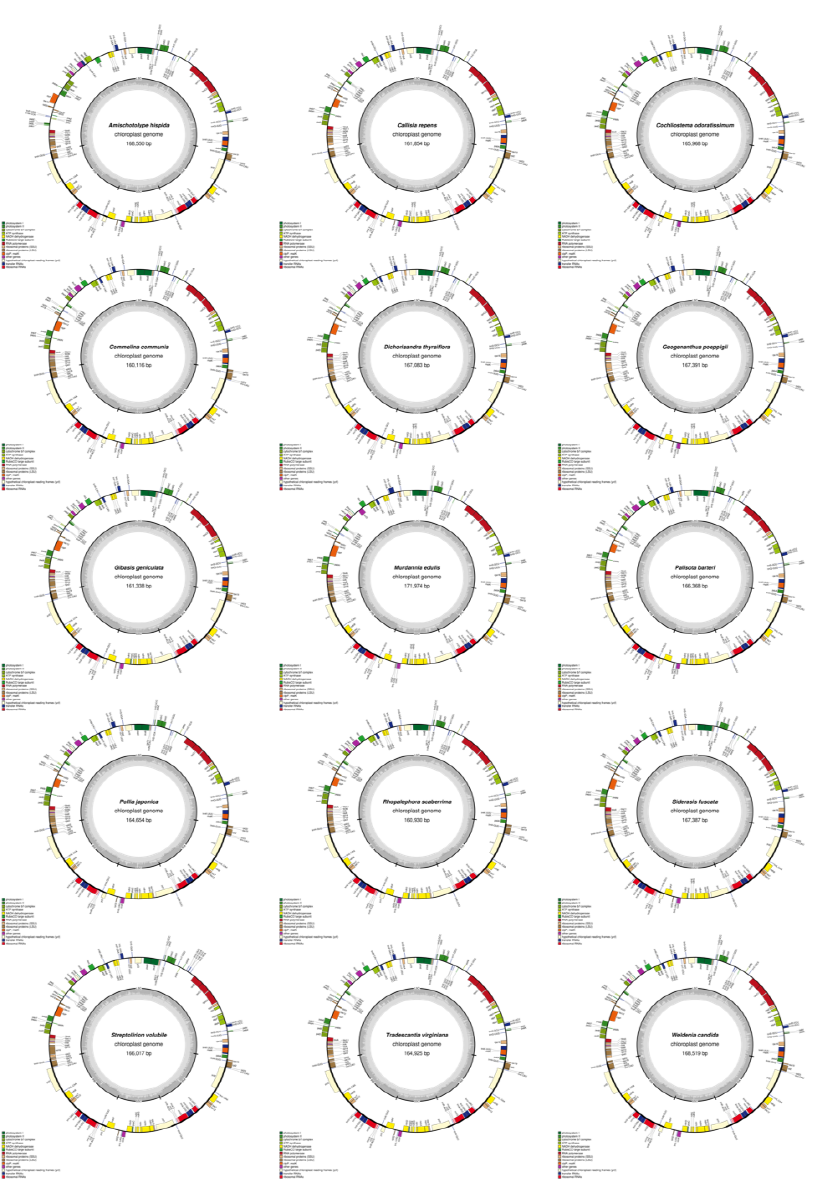

Supplement: Supplementary file 1 — Additional file 1: Table S1. List of sampling taxa from 15 species of Commelinaceae and assembly information. Table S2. List of species used for phylogenomic analyses. Table S3. Nucleotide diversity (Pi) of 16 Commelinoideae species. Figure S1. Complete chloroplast genome of 15 Commelinaceae taxa in this study. Figure S2. Amino acid alignment of plastid accD and rpoA genes within 22 Commelinales taxa. [file 12864_2021_7541_MOESM1_ESM.zip › Figure S1.png]

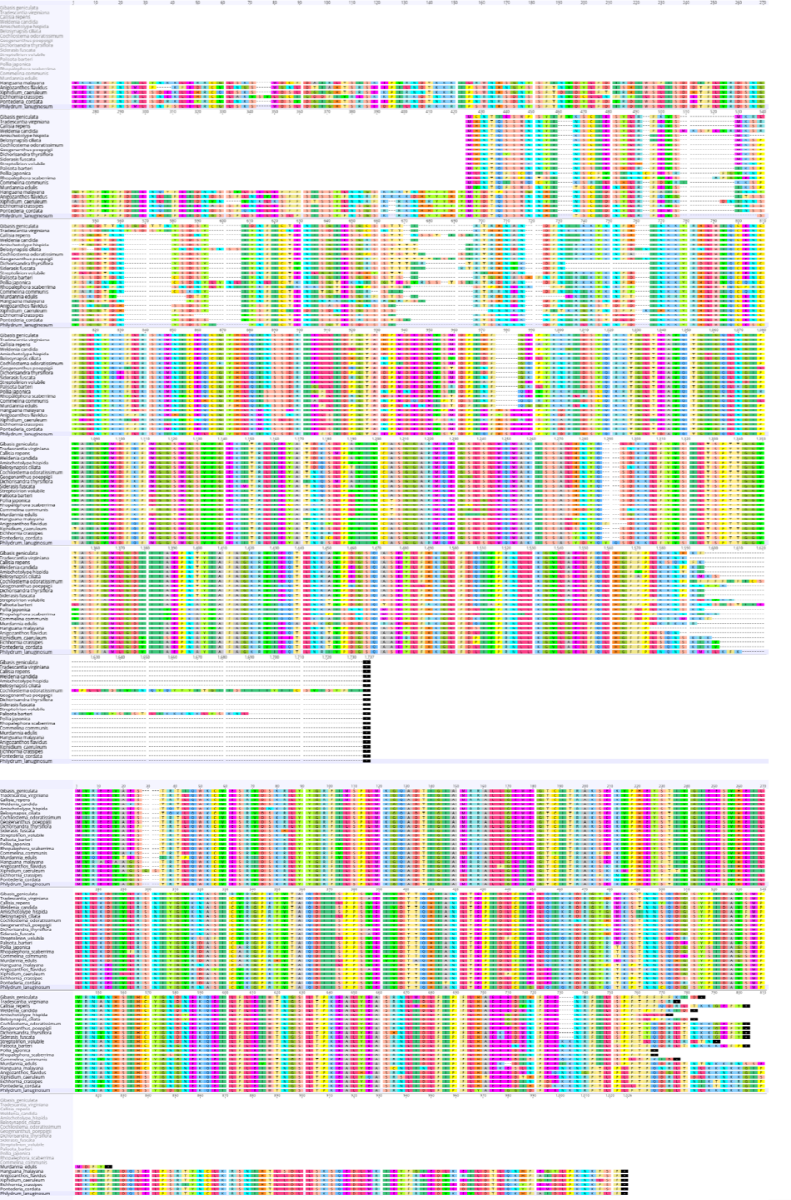

Supplement: Supplementary file 1 — Additional file 1: Table S1. List of sampling taxa from 15 species of Commelinaceae and assembly information. Table S2. List of species used for phylogenomic analyses. Table S3. Nucleotide diversity (Pi) of 16 Commelinoideae species. Figure S1. Complete chloroplast genome of 15 Commelinaceae taxa in this study. Figure S2. Amino acid alignment of plastid accD and rpoA genes within 22 Commelinales taxa. [file 12864_2021_7541_MOESM1_ESM.zip › Figure S2.png]
